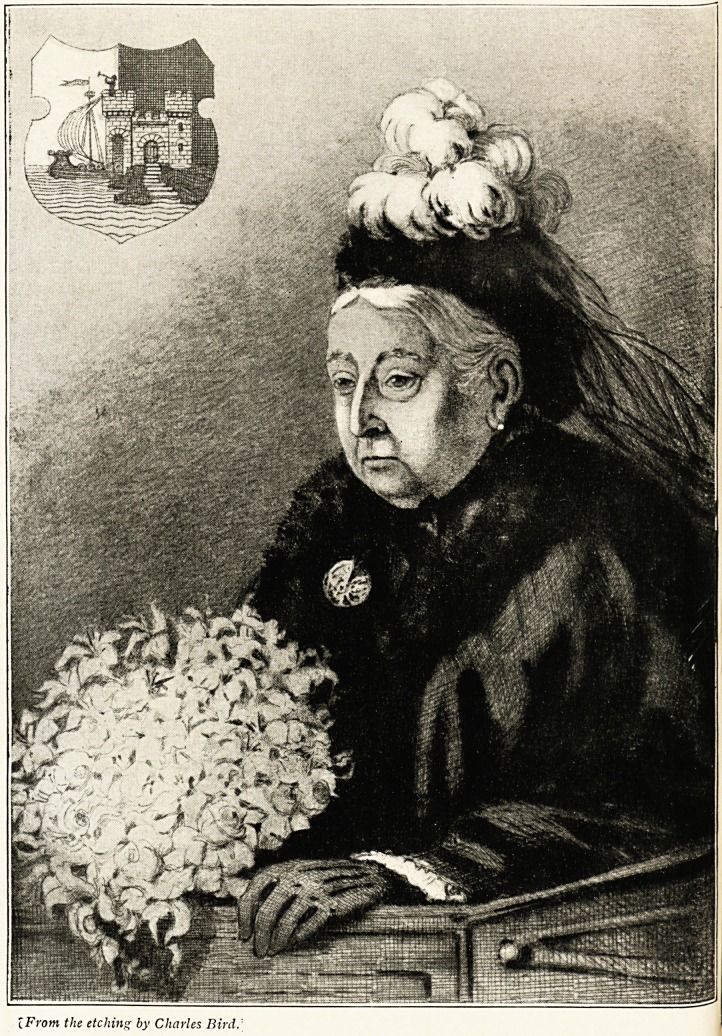# In Memoriam

**Published:** 1901-03

**Authors:** 


[*From the etching by Charles Bird.*]

The Bristol

Medico-Chirurgical Journal.

MARCH, 1901.

### In Memoriam.

As a frontispiece to our first volume of the new century we present to our subscribers a reproduction of an excellent portrait of the revered and beloved Queen Victoria. It represents Her late Majesty at the time of her visit to Bristol at the opening of the Queen Victoria Jubilee Convalescent Home, on November 15th, 1899. She is depicted as sitting in the carriage and attentively listening to the address which was then being read to her. The occasion is a memorable one in Bristol history as being that on which the first Lord Mayor of this city, Sir Herbert Ashman, received the honour of knighthood on the steps of the Council House. The city arms in the top corner, and (in the original etching) an outline sketch of the Corporation casket presented to the Queen, with the names of the Lord Mayor, High Sheriff, Aldermen and Councillors, give the portrait an interest locally, as also does the fact that the picture is the work of our well-known etcher, Charles Bird, whose various productions deservedly receive the highest commendation. Of the numerous representations of Her Majesty which have been published, we think that this one should take a very high place.

## Figures and Tables

**Figure f1:**